# Industrial-age doubling of snow accumulation in the Alaska Range linked to tropical ocean warming

**DOI:** 10.1038/s41598-017-18022-5

**Published:** 2017-12-19

**Authors:** Dominic Winski, Erich Osterberg, David Ferris, Karl Kreutz, Cameron Wake, Seth Campbell, Robert Hawley, Samuel Roy, Sean Birkel, Douglas Introne, Michael Handley

**Affiliations:** 1 Department of Earth Sciences, Dartmouth College, Hanover, NH 03755 USA; 2 Climate Change Institute and School of Earth and Climate Sciences, University of Maine, Orono, Maine 04469 USA; 3 Institute for the Study of Earth, Oceans, and Space, University of New Hampshire, Durham, NH 03824 USA

## Abstract

Future precipitation changes in a warming climate depend regionally upon the response of natural climate modes to anthropogenic forcing. North Pacific hydroclimate is dominated by the Aleutian Low, a semi-permanent wintertime feature characterized by frequent low-pressure conditions that is influenced by tropical Pacific Ocean temperatures through the Pacific-North American (PNA) teleconnection pattern. Instrumental records show a recent increase in coastal Alaskan precipitation and Aleutian Low intensification, but are of insufficient length to accurately assess low frequency trends and forcing mechanisms. Here we present a 1200-year seasonally- to annually-resolved ice core record of snow accumulation from Mt. Hunter in the Alaska Range developed using annual layer counting and four ice-flow thinning models. Under a wide range of glacier flow conditions and layer counting uncertainty, our record shows a doubling of precipitation since ~1840 CE, with recent values exceeding the variability observed over the past millennium. The precipitation increase is nearly synchronous with the warming of western tropical Pacific and Indian Ocean sea surface temperatures. While regional 20^th^ Century warming may account for a portion of the observed precipitation increase on Mt. Hunter, the magnitude and seasonality of the precipitation change indicate a long-term strengthening of the Aleutian Low.

## Introduction

The regional precipitation response to anthropogenic forcing is an area of intense research because of potential societal impacts through changes in mean hydroclimate state and the frequency and magnitude of floods and drought^1,2^. General circulation models (GCMs) project an overall 1.5–2% increase in global precipitation per degree of warming and an increased contrast between wet and dry areas^3^. However, the hypothesized precipitation increase and regional wet-dry patterns are equivocal in the relatively short instrumental record, particularly over land^4^.

One region that has experienced significant 20^th^ century precipitation change is the North Pacific, where wintertime precipitation is largely governed by the strength of the Aleutian Low. A stronger Aleutian Low is associated with enhanced northward flow of relatively warm, moist air masses into Alaska, causing increased wintertime precipitation along the southern Alaska coast^5^. From 1950 to 2011, many coastal Alaskan weather stations experienced significant increases in winter precipitation (Juneau: +40%; Kodiak: +67%; Palmer: +36%; Seward: +35%; Yakutat: +26%)^6^ in concert with a strengthening Aleutian Low. However, the Aleutian Low has strong interannual to multidecadal variability^5^ including a state shift in 1976^7,8^, so the short observational period (60–65 years) limits our ability to assess secular trends in regional precipitation^9^. A critical but unresolved question is whether the recent changes in the Aleutian Low and North Pacific precipitation are within the range of natural interannual to decadal-scale climate variability.

**Figure 1:**
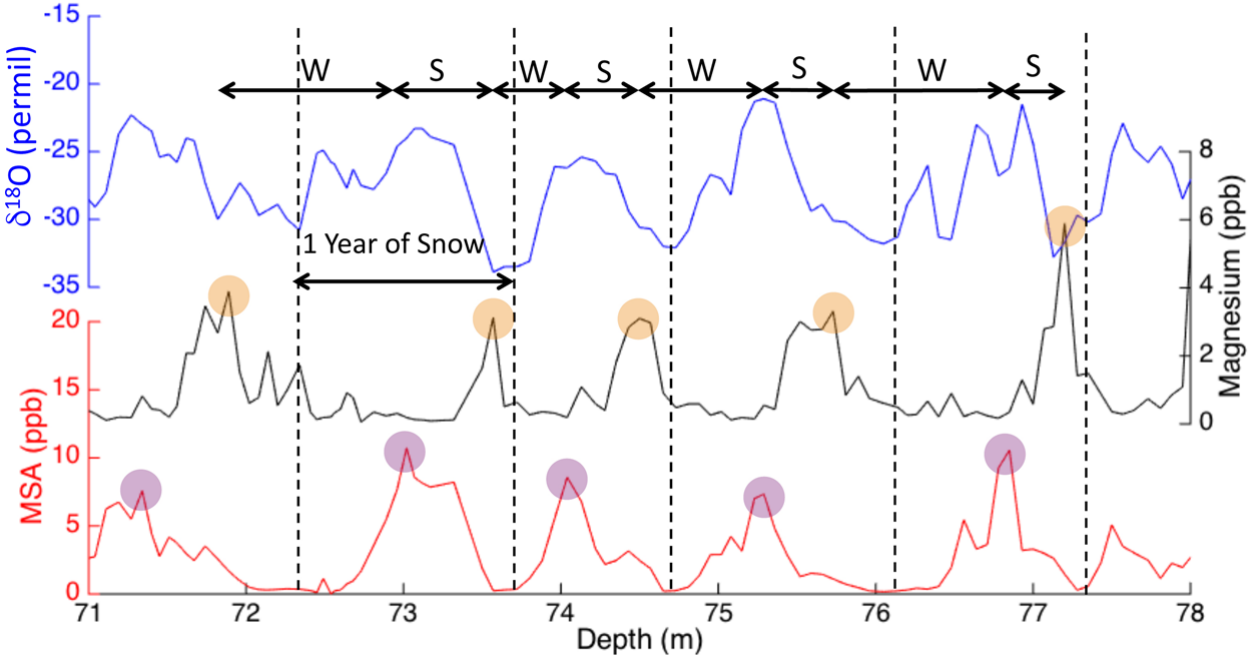
Annual layer counting in the Mt. Hunter ice core. (A) Three chemical series exhibiting annual layers are shown at a representative depth of core: Mg (black), δ^18^O (blue) and MSA (red). Each vertical dotted line represents the depth of Jan. 1^st^ in a δ^18^O trough and just below a Mg peak. The distance between each vertical dotted line represents one year’s snow accumulation (before thinning correction). The position of these years was selected three times by three independent researchers. We delineate summer (May-August) and winter (September-April) seasons by recording the late summer-fall peak positions of MSA (purple circles) and the spring peak positions of Mg (orange circles).

Here, we present a seasonally- to annually-resolved 1200-year ice core record of North Pacific precipitation to provide long-term context for the observed trend since the 1950s, and to evaluate forcing mechanisms. Our record is derived from two ice cores (each 208 m long) collected from the Mt. Hunter summit plateau (62**°**56′N, 151**°**5′W, 3900 m) in Denali National Park, Alaska. A high snow accumulation rate (1.15 m water equivalent [w. e.] average since 1900) and infrequent surface melt (<0.5% of the core is composed of refrozen melt layers and lenses) at the Mt. Hunter drill site preserve robust seasonal oscillations of several chemical parameters (Na, Ca, Mg, NH_4_^+^, MSA, δ^18^O, liquid conductivity, dust concentration), facilitating annual layer counting back to 800 CE (Fig. 1).

All ice core accumulation rate records are subject to progressive annual layer thinning at depth due to glacier flow^10^, which must be corrected to determine the true accumulation-rate time series. We correct for layer thinning using four ice-flow models to obtain a conservative estimate of uncertainty in the Denali accumulation record^11–13^. One of the models is a 3-dimensional finite element model from Campbell *et al*.^14^ (hereafter the “Campbell model”), which is derived from the well-characterized basin geometry and surface ice velocities (determined by extensive geophysical surveys) at the Mt. Hunter drill site^14^. The other models are based on approaches from sites where previously published ice core accumulation rates have been reconstructed. We vary the model inputs such that each of the four models includes multiple simulations representing the entire range of plausible geophysical conditions at the drill site. Specific details on the mechanics of each thinning model, the exploration of a range of plausible thinning scenarios and the quantification of model uncertainty can be found in the Supplemental Information. While previous alpine ice core accumulation records^15–21^ are based on only one flow model for thinning corrections, the use of four different flow thinning models, the extensive characterization of drill site geometry and geophysical attributes^14^, careful quantification of timescale uncertainty, and the use of two ice cores from the same drill site, combine to significantly increase our confidence in the veracity of the Denali accumulation record. Critically, the four different flow models all produce similar trends in accumulation since 1500 CE.

## Results

**Figure 2:**
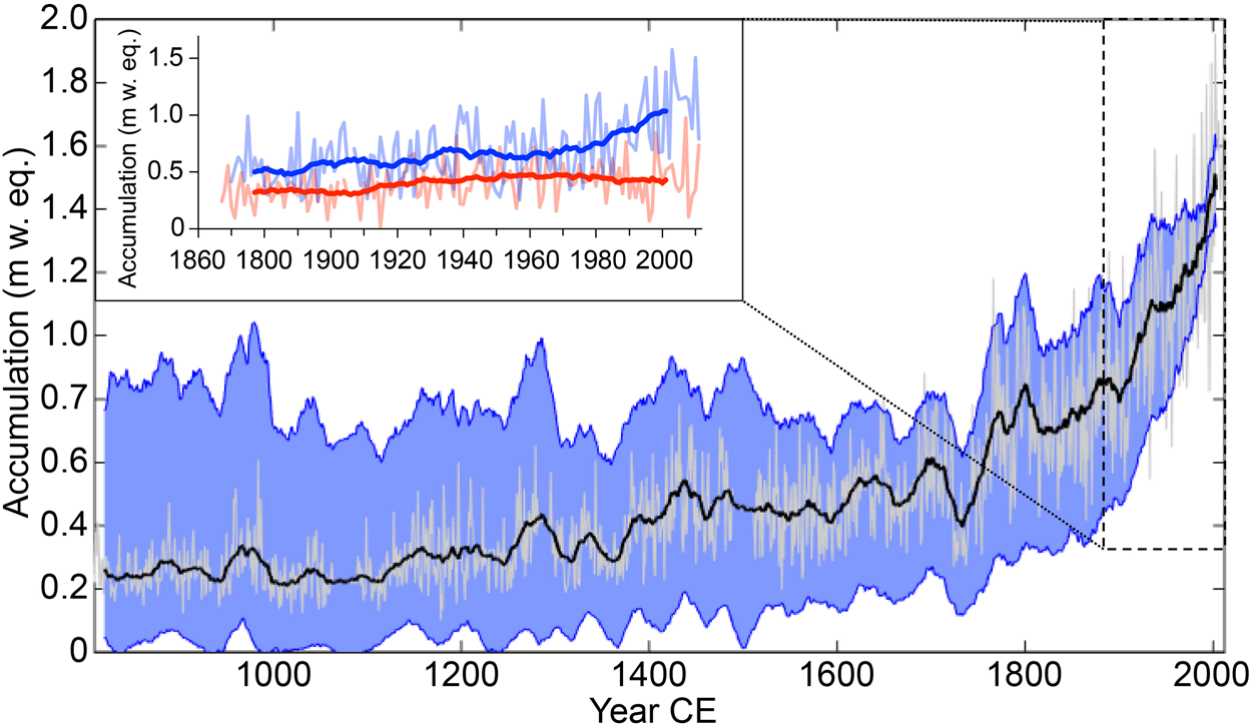
The Mt. Hunter accumulation record. Annual (light gray line) and 21-year smoothed (black line) accumulation time series from the year 810 CE to present, constrained by 21-year smoothed error envelopes (blue shading) inclusive of stochastic, peak position and layer-thinning model uncertainties (see Methods section), including the total uncertainty range among all four modeling approaches. The inset shows seasonal trends in accumulation since 1867 with 21-year running means (bold lines). Snowfall accumulating between September and April (blue) has more than doubled, with a faster rise since 1976. Summer accumulation (April to August; red) remained comparatively stable except for a baseline shift between 1909 and 1925.

**Figure 3:**
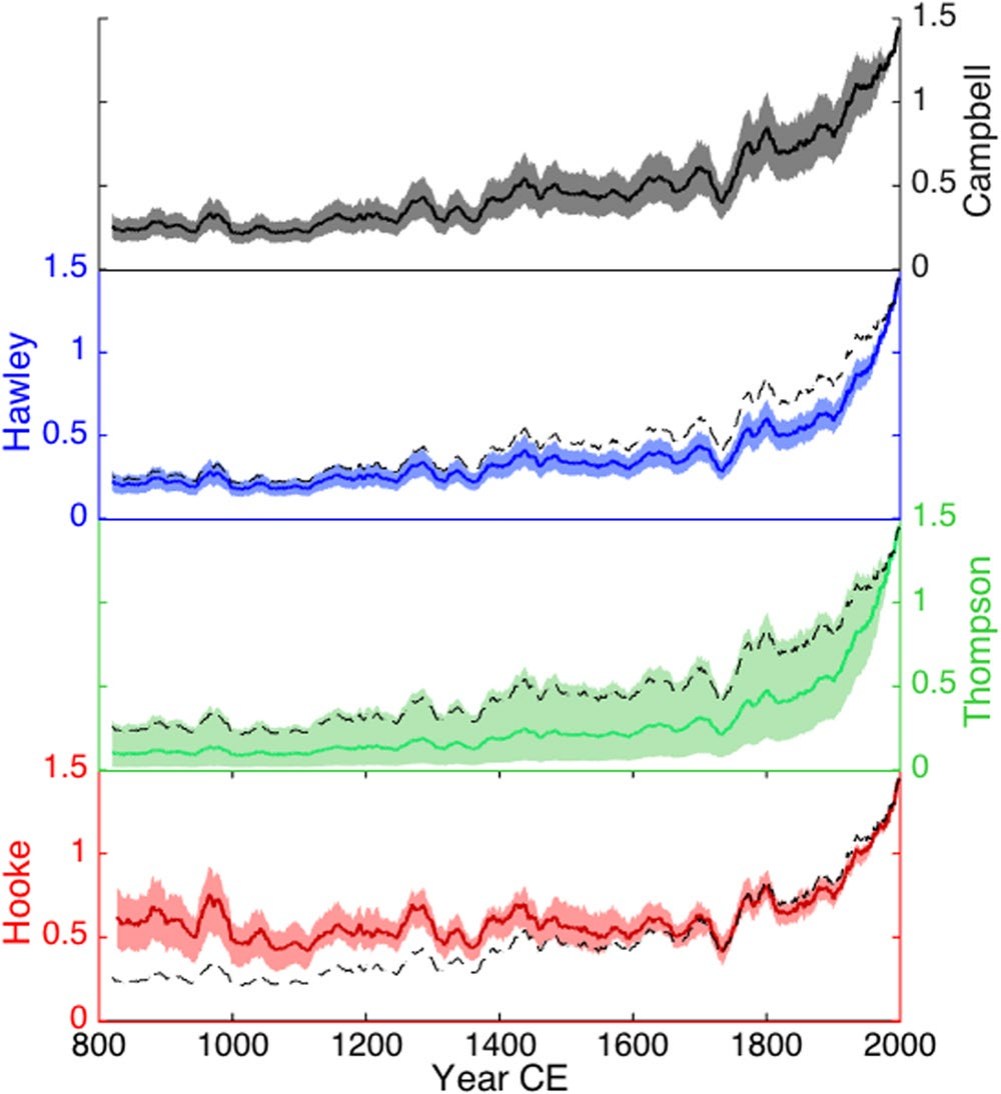
Accumulation rate time series from 810 CE to the present after correction by four independent layer-thinning model approaches: Campbell^14^ (black), Hawley (blue), Thompson^13,18–20^ (green) and Hooke^12^ (red); see supplementary information for details about each. Y-axes are equivalent in each panel, ranging from 0 to 1.5 meters of water equivalent annual accumulation. Results are smoothed with a 21-year running mean for clarity. Shading indicates the full range of accumulation estimates produced among different scenarios in each model. The central estimate in the Campbell model is shown for comparison in the bottom three panels as a dotted line. Every model shows unprecedented levels of snow accumulation in the 20^th^ century under any set of plausible input parameters.

The annually resolved Denali snow accumulation record (Fig. 2) indicates that the post-1950 precipitation increase in the Alaskan weather station records began well before the 20^th^ century, in circa 1840 CE. The Campbell model produces the smallest accumulation increase since 1840 (Fig. 3), with a doubling of the median accumulation rate between 1600–1840 (0.58 m a^−1^) and 1950–2013 (1.29 m a^−1^), and an average increase of 9.6 mm a^−2^ over the later period (Figs 2, 3). The first 700 years (800–1500 CE) of the record are characterized by multi-decadal variability and larger uncertainties among our thinning model ensemble that allow for a declining or rising long-term accumulation trend from 800–1500 CE (Fig. 3). Despite the larger uncertainties before 1500 CE, the Denali record shows that accumulation rates from 1950–2013 were the highest of the last 1200 years under all plausible conditions of glacier flow or layer counting uncertainty (Figs 2 and 3). There is nearly complete agreement between the accumulation records in the two Denali ice cores, with a correlation coefficient of 0.92 (p < 0.001). Here, and throughout the text, p-values are adjusted to account for the reduced effective sample size resulting from autocorrelation.

From 1867–2013 CE (133 m depth), the Denali accumulation record can be confidently separated into summer (May-August) and winter (September-April) accumulation due to the high sampling resolution and thick annual layers (Fig. 2 inset). This summer-winter parsing is performed using spring peaks in magnesium (from dust) and late summer-fall peaks in methanesulfonic acid (MSA), which have consistent offsets from other seasonal indicators including ammonium (summer) and δ^18^O (summer peak, winter trough; see Methods; Fig. 1). Figure 2 inset shows that the recent doubling of annual accumulation is dominated by a wintertime increase of 3.6 mm a^−2^ since 1867, representing an increase of 117% over this interval. The increase in Denali winter accumulation is highly significant (p < 0.001) in both Denali ice cores based on comparison of the winter means from 1870–1900 and 1980–2010, and a non-parametric Thiel-Sen robust linear regression^22^.

**Figure 4:**
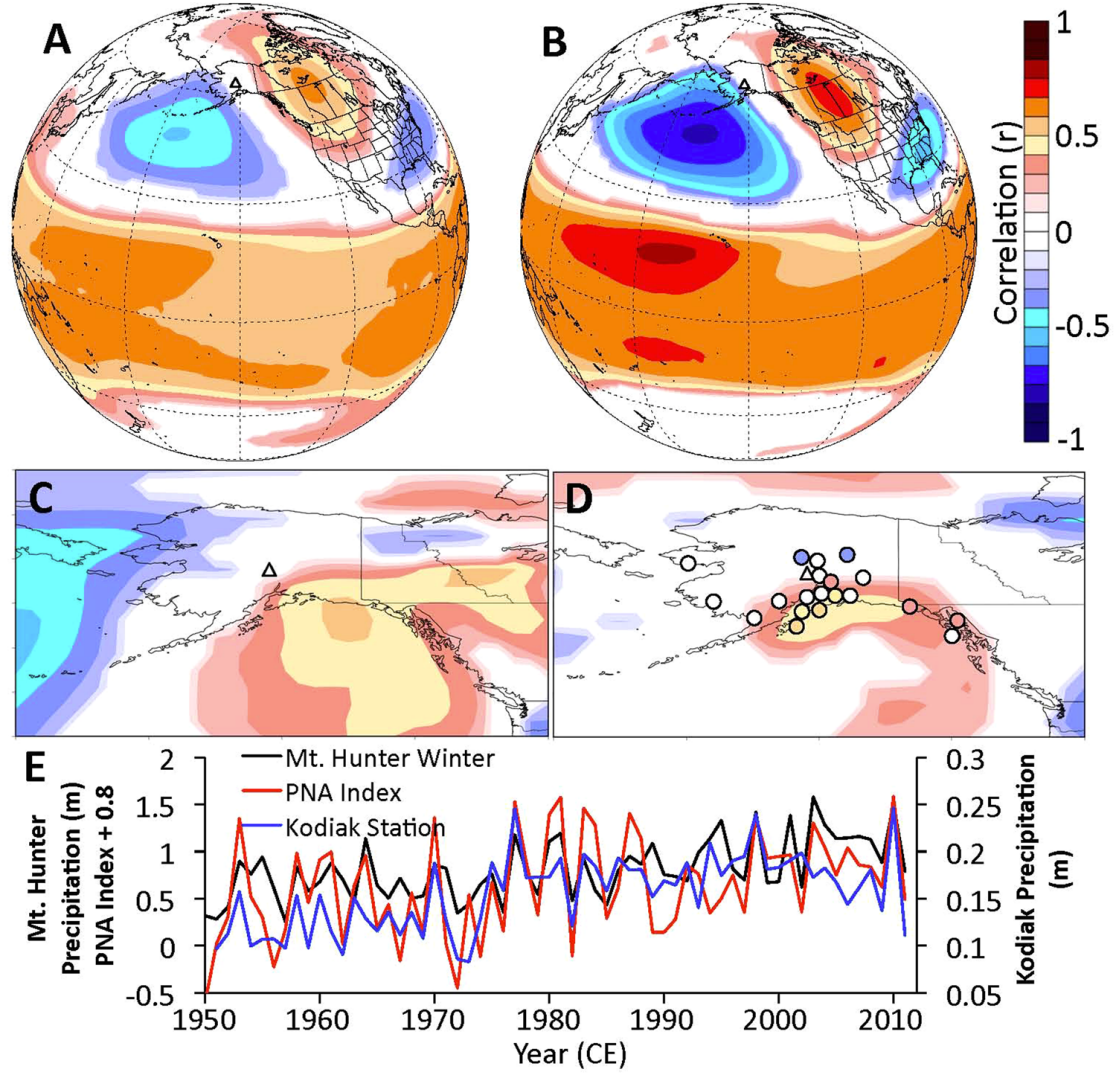
Climatological correlations with winter accumulation on Mt. Hunter. (**A**–**D**) Spatial Pearson correlations between (**A**) 20^th^ Century reanalysis^27^ DJF 500 mb geopotential heights and Mt. Hunter (triangle) winter accumulation from 1910–2010; (**B**) reanalysis DJF 500 mb geopotential heights and September-April PNA index from 1950–2010; (**C**) reanalysis DJF surface meridional winds and Mt. Hunter winter accumulation; (**D**) Mt. Hunter winter accumulation and reanalysis DJF precipitation (color shading) and September-April precipitation recorded at Alaskan weather stations (color-filled circles). Colors only filled in areas exceeding 90% significance. (**E**) Time series of Mt. Hunter winter accumulation (black), the PNA index (red), and Kodiak precipitation (blue) for September-April from 1950–2011. Images obtained using Climate Reanalyzer (http://cci-reanalyzer.org), Climate Change Institute, University of Maine, USA.

Winter Denali accumulation is highly and significantly correlated with winter precipitation at several southern coastal Alaska weather stations, including Valdez, Yakutat, Seward, Juneau, Homer, and Kodiak (Table 1, Fig. 4). The significant correlations (p < 0.05) with Alaskan station data provide additional confidence in our seasonal parsing and overall fidelity of the Denali accumulation record. Furthermore, the south coastal Alaska station records and the wintertime ice core accumulation record all show the distinct 1976/77 step increase in precipitation associated with the PDO transition from a negative to a positive mode^8,9,23,24^ (Fig. 4E). However, while the weather station records show a step increase in precipitation during the late 1970s, the Denali accumulation record shows that winter precipitation continued to rise on Mt. Hunter after the 1976 shift. We revisit this point in the discussion section below.

**Table 1:** Correlations between accumulation data in the Mt. Hunter ice core and precipitation at regional meteorological stations. Correlation values (r) between the ice core and meteorological station data are calculated annually, during summer (MJJA), and during winter (SONDJFMA) over their respective periods of record. Values significant to 95% are printed in bold. Data obtained using Climate Reanalyzer (http://cci-reanalyzer.org), Climate Change Institute, University of Maine.

Station	Number of Years	Annual	Summer	Winter
Anchorage	60	**0.27**	0.25	0.23
Fairbanks	64	0.01	0.13	**−0.31**
Talkeetna	94	0.12	**0.30**	**−**0.06
Cordova	63	0.15	0.11	0.19
Gulkana	67	0.04	0.08	0.00
Juneau	63	0.25	−0.06	0.38
Kodiak	63	**0.47**	0.07	**0.59**
Sitka	60	0.02	0.03	0.17
Valdez	95	**0.25**	**−**0.10	**0.40**
Yakutat	95	0.12	**−**0.07	**0.30**
King Salmon	84	0.09	0.06	0.02
Tanana	94	**−**0.01	0.15	**−0.30**
Bethel	88	**0.23**	0.22	**−**0.03
Nome	63	0.13	0.15	0.14
Homer	81	**0.33**	0.20	**0.43**
Iliamna	70	**0.36**	**0.36**	0.20
Kenai	68	0.13	**0.34**	0.14
McKinleyPark	88	0.07	0.09	**−**0.16
Palmer	63	**0.46**	0.14	**0.31**
Seward	103	**0.44**	**0.24**	**0.55**

Summer accumulation in the Denali record has a more muted but still significant (p < 0.001) increase of 1.2 mm a^−2^ since 1867, or a total rise of 49% over this interval (Fig. 2). Correlations between the ice core record and Alaskan stations during summer months are restricted to nearby stations in South Central Alaska, including Talkeetna, Homer, Bethel, Iliamna, Kenai, and Seward (Table 1). The summer increase also has a different timing from the wintertime record, characterized by an increase between 1905 and 1925 and relative stability throughout the remainder of the record (Fig. 2 inset). Because of the high temporal resolution (≥8 samples/year) during the last century, the timing of the increases in summer and winter precipitation are robust to seasonal partitioning.

Central Alaska has warmed by 2–3° since 1950^8^, and higher precipitation is expected from higher temperatures through the Clausius-Clapeyron (C-C) increase in absolute humidity^3^. While likely responsible for some of the precipitation increase on Mt. Hunter, the observed warming is insufficient to account for most of the doubling of precipitation recorded in the Denali ice core, even with enhanced (14% K^−1^) C-C scaling rates over mountainous regions^25^.

It is very likely that that a strengthening Aleutian Low is responsible for the majority of the accumulation increase observed in the Mt. Hunter ice core. This is supported by the strong relationship between winter Denali accumulation and winter precipitation recorded in weather stations along the Gulf of Alaska, which are sensitive to changes in Aleutian Low strength^26^. Wetter winters in the Mt. Hunter ice core record between 1910–2010 were significantly associated with a deeper Aleutian Low at the surface (p < 0.05; Fig. S4) and 500 mb level (p < 0.1; Fig. 4A) in NCEP/NCAR 20^th^ century reanalysis data^27^. The 500 mb anomalies include a downstream enhanced ridge over western North America and deeper low over the southeast United States characteristic of the PNA positive teleconnection pattern^28^ (Fig. 4A–B). The correlation between the PNA index and winter Denali accumulation from 1950 to 2010 is r = 0.64 (p < 0.0001; Fig. 4E), consistent with a strong relationship between increasing Mt. Hunter snow accumulation and an intensifying Aleutian Low. A 20^th^ century strengthening Aleutian Low is also supported by a significant negative trend in the North Pacific Index (NPI), a sea-level pressure index over the Aleutian Low region^29^. The negative trend in NPI index during SONDJFMA is significant to p < 0.02, and during DJF is significant to p < 0.01. Previous work indicates that the strength of the Aleutian Low tends to co-vary with its mean position, with a stronger (weaker) Aleutian Low shifted towards the east (west)^30,31^. However, our observations show that the highest accumulation winters on Mt. Hunter are associated with a sea level pressure anomaly centered over the Aleutian Islands (Fig. S4). Thus, we observe no strong evidence for covariance between Aleutian Low position and Mt. Hunter snowfall.

## Discussion

Under all four ice-thinning models, the Mt. Hunter snow accumulation rates in the 20^th^ century are unprecedented in the last millennium (Figs 2, 3). The Campbell finite element ice flow model^14^ produces the highest degree of thinning since 1650 CE among any of the model simulations we used, and therefore is the most conservative model in that it produces the smallest precipitation increase on Mt. Hunter since 1840 CE. This is our preferred ice flow model because it is constrained by the detailed basin and glacier geometry, spatial accumulation patterns, and surface ice velocity of the Mt. Hunter ice core drill site. Prior to 1650, the Hooke model^12^ consistently produces higher accumulation values than the other thinning models (Fig. 3). This may be because parameters in the Hooke model are statistically optimized to match the timescale, as opposed to the other three models where thinning corrections are based on explicit calculations of vertical strain rate (see Supplemental Information). Even in the Hooke model, the accumulation rates before 1840 never approach 20^th^ century values (Fig. 3).

Numerous accumulation reconstructions from alpine ice cores have been published during recent decades. Mt. Hunter is very similar to many of these sites with low velocities near the drill site (approximately 2 m a^−1^), surface-conformable stratigraphy, and very low surface slope. The average annual temperature on Mt. Hunter (−17 °C) is cooler than at most other alpine ice core sites but is similar to Ushkovsky volcano^32^ (−15.7 °C) and Dasuopu glacier^33^ (−16 °C). Many other alpine ice core accumulation records have been drilled at sites with saddle-like geometry including Aurora Peak^16^, Mt. Waddington^21^, Mt. Everest^12^, Belukha Saddle^34^ and Mt. Logan^15^. Thus, we would expect that the modeling approaches published in these studies would be applicable to the conditions on Mt. Hunter. Nevertheless, we use four different modeling approaches, one of which is designed for the specific Mt. Hunter site geometry, and all of which have very conservative and rigorously quantified ranges of uncertainty (Fig. 3 and Supplemental Information). To our knowledge, no other alpine ice core accumulation record has been developed with such a thorough characterization of the thinning regime or uncertainties. All of the thinning models produce a robust increase in accumulation since the mid-19^th^ century above late-Holocene background values.

While all of our modeling simulations agree that during the early half of our record (810–1400 CE) precipitation remained well below present values, we cannot confidently conclude from our results whether precipitation was increasing or decreasing within this time period. One simulation (the Hooke model^12^) shows a slight decrease in accumulation rate between 810 and 1400 CE while the remaining three modeling approaches suggest a slight accumulation increase during this period with superimposed decadal to centennial scale variability. Relatively low snow accumulation on Mt. Hunter between 1000 and 1300 CE is consistent with low sodium concentrations in the Mt. Logan and Mt. Hunter records during this interval, indicative of a relatively weak Aleutian Low (Fig. S5)^35,36^.

We suggest that the recent doubling of Alaska Range snowfall recorded in the Denali ice core reflects an increase in absolute humidity with warming and an intensification of the Aleutian Low to its strongest levels (most positive PNA) of the past millennium. The precipitation increase we observe on Mt. Hunter is apparent in other Alaskan records. Between the mid-20^th^ century and present day, several weather stations along coastal Alaska^6^ (e.g. Kodiak, Palmer, Juneau) have experienced similar (but smaller) increases in wintertime precipitation compared with Mt. Hunter (Fig. S6). Consistent with our results, a century-long ice core collected from Aurora Peak in the eastern Alaska Range^16^ (240 km from Mt. Hunter) also records a doubling of snow accumulation over the 20^th^ century, with greater rates of precipitation increase than observed on Mt. Hunter (Figs S5, S6). The Mt. Logan ice core (Yukon, Canada) time series of accumulation, sodium, and water isotope ratios show trends over the past 200 years indicative of a strengthening Aleutian Low^15,35,37^ (Fig. S5). Specifically, Moore *et al*.^15^ found a positive and accelerating trend in Mt. Logan annual accumulation after the middle of the nineteenth century, and interpret it as indicative of a progressively more positive PNA. This was supported by Rupper *et al*.^38^ who confirm that high accumulation years on Mt. Logan are associated with a stronger wintertime Aleutian Low. Furthermore, the composite Mt. Logan and Mt. Hunter sea-salt Na^+^ calibrated Aleutian Low proxy similarly supports a progressive strengthening of the Aleutian Low over recent centuries to its strongest levels of the past 1500 years^36^ (Fig. S5).

The doubling of accumulation rates at Mt. Hunter, Aurora Peak and other sites near the Gulf of Alaska stand seemingly in contrast to the more modest 2–3% increases in precipitation per degree of warming predicted by climate models (e.g. Held and Soden^3^). The rapid precipitation increase in Alaska represents a regional signal amplified primarily by enhanced advection of moisture and storms into the Gulf of Alaska associated with a strengthening Aleutian Low. With a deeper Aleutian Low, the largest precipitation increases would be expected to occur where southerly flow on the eastern side of the low encounters the Alaskan coastline and mountain ranges. This is exactly the pattern we observe, with increasing precipitation at coastal Alaskan weather stations including Kodiak, Juneau and Valdez, and the strongest precipitation increases at orographic barriers such as Mt. Hunter and Aurora Peak (Fig. 4d; Fig. S6). Previous work has shown that this configuration leads to drier conditions within the rain shadow of these orographic barriers^39^, which is apparent as negative correlations between winter Mt. Hunter accumulation and precipitation at Tanana, McKinley Park, and Fairbanks located north of the Alaska Range (Table 1). Additionally, Tanana winter precipitation has experienced a significant decrease since 1950^6^. We do not, therefore, infer that the Mt. Hunter precipitation increase is representative of the expected broader mid-high latitude precipitation increase predicted by global scale climate projections^40^. Rather, the Mt. Hunter record captures a specific regional phenomenon (a deepening Aleutian Low) that has occurred in conjunction with warming, resulting in a dramatic increase in regional precipitation. Finally, ice core^17^ and other paleoproxy records^41^ from outside the North Pacific region do not document long-term precipitation increases, consistent with our finding that the North Pacific hydroclimate intensification is a regional feature associated with a strengthening Aleutian Low.

**Figure 5:**
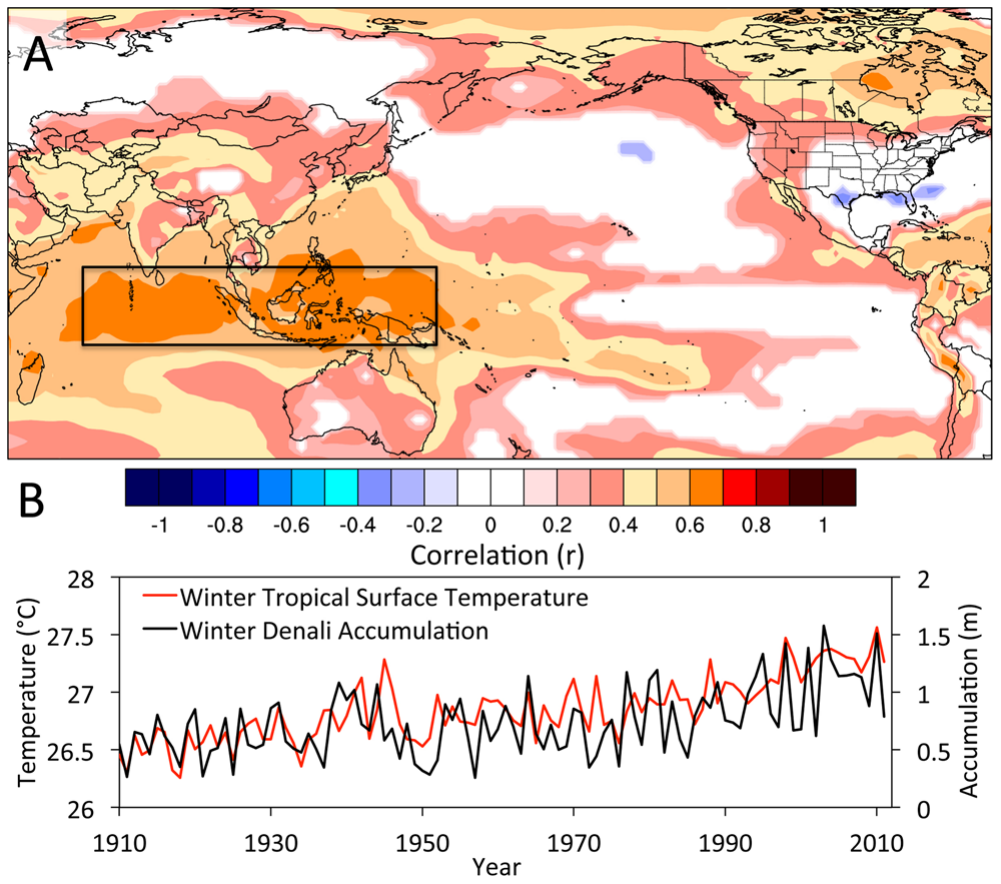
Relationship between tropical Pacific and Indian Ocean temperatures and accumulation on Mt. Hunter (triangle). (**A**) Spatial correlation between annual Mt. Hunter accumulation and annual surface temperature. (**B**) Time series of wintertime western tropical Pacific and Indian Ocean temperatures compared to Mt. Hunter wintertime accumulation. 20th Century Reanalysis V2 data^27^ from 1910–2010 is used in each panel. The black box delineates the Indian and western tropical Pacific Ocean region averaged in panel B. Colors only filled in areas exceeding 90% significance. Images obtained using Climate Reanalyzer (http://cci-reanalyzer.org), Climate Change Institute, University of Maine, USA.

Dynamical studies suggest that the most plausible way to intensify the Aleutian Low is through warmer tropical SSTs, particularly in the western tropical Pacific and Indian Oceans^42^. Enhanced convective precipitation and latent heat release associated with higher tropical Pacific and Indian Ocean SSTs induce a Rossby wave extratropical response manifesting as the PNA positive pattern^42,43^ (Fig. 4b). Consistent with this dynamical theory, the annual Mt. Hunter accumulation record from 1910–2010 is significantly correlated (r = 0.59, p < 0.01) with reanalysis SSTs in the western tropical Pacific and Indian Oceans (Fig. 5), supporting a strong link between Alaskan precipitation and tropical SSTs. This high correlation is due in part to the highly trended nature of both records, with warming temperatures over the tropical Pacific and Indian Oceans and rising accumulation in the Alaska Range. However, a significant correlation remains after detrending both time series (r = 0.39, p > 0.01), demonstrating the robust nature of the link between the tropical Pacific and Indian Ocean and North Pacific climate identified by previous studies^42,43^.

**Figure 6:**
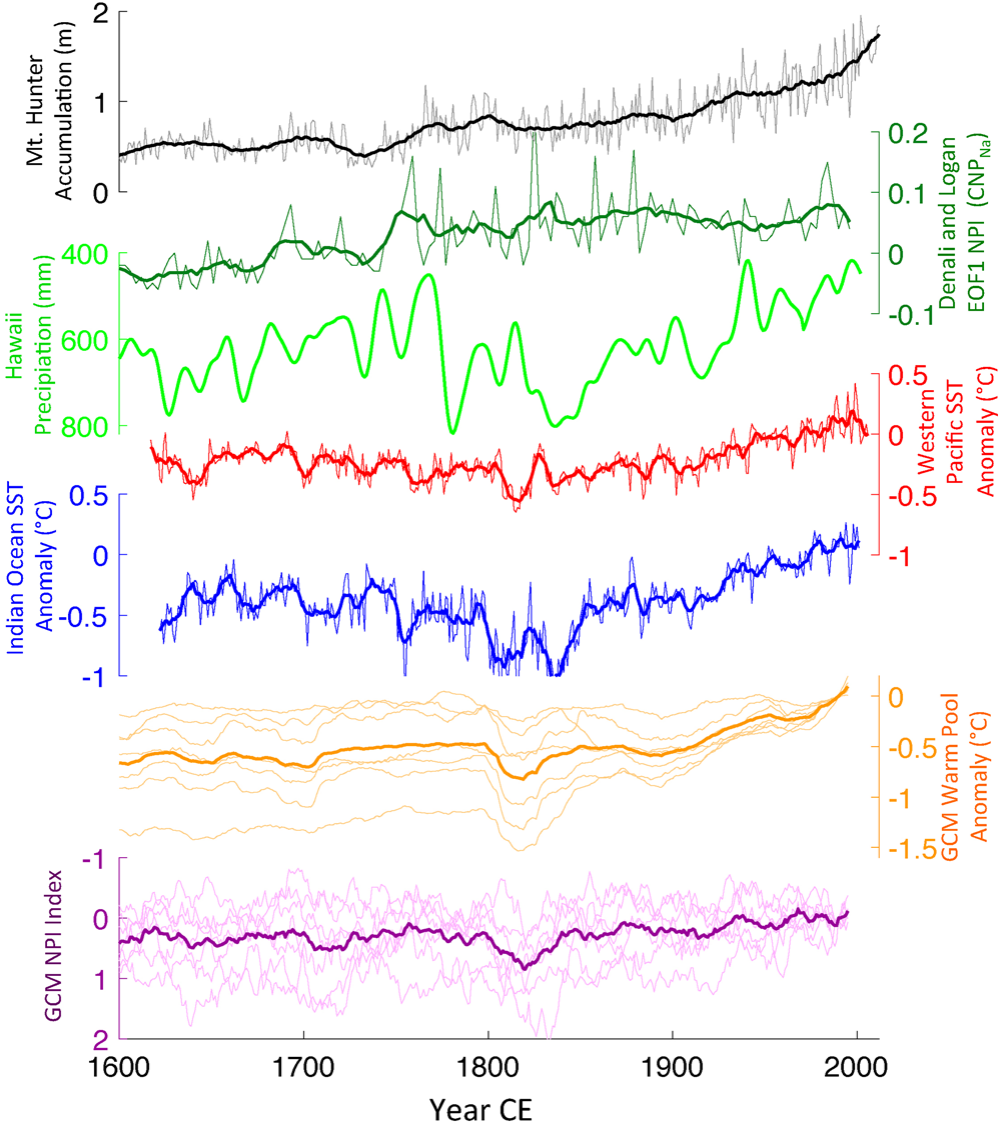
Paleoclimate context for Mt. Hunter precipitation increase. The Mt. Hunter annual accumulation time series (light grey) with a 21-year running mean (solid black) compared with reconstructed Hawaii rainfall^46^ (green), Western Pacific (red) and Indian Ocean (blue) SST anomalies^44,45^, and 21-year running mean GCM reconstructions of Western Pacific temperature anomalies (orange), and the NPI index (purple) from the CMIP5 Last Millennium experiments. With the exception of the GCM reconstructed NPI index, each dataset shows a sustained directional trend from the early 1800s to the present.

In Fig. 6, we compare the Denali accumulation record to multiproxy records of SSTs in the Western Pacific and Indian Oceans^44,45^. Sustained and significant changes since ~1840 CE are evident in each dataset (Denali = 1840 CE, Western Pacific = 1834 CE, Indian = 1827 CE), which we confirm using the change detection methods described in Abram *et al*.^44^ (see Methods). This is consistent with the doubling of Denali snow accumulation since ~1840 being forced, at least in part, by warmer western Pacific and Indian Ocean SSTs through the atmospheric bridge^43^. The secular increase in Denali and Mt. Logan ice core sea salt Na^+^ concentrations over recent centuries has also been interpreted as an extratropical response to warming Pacific SSTs^35,36^, as has a dramatic increase in the Mt. Logan δ^18^O record at 1840^37^ (Fig. S5). Similarly, a proxy record of Hawaii precipitation includes a secular change towards more PNA positive conditions, reflected as a long-term precipitation decrease (Fig. 6) associated with anomalously high pressure in the subtropical North Pacific^46^ (Fig. 4b). Thus, through the atmospheric teleconnection, warmer western tropical Pacific and Indian Ocean waters have supported an increase in Alaskan precipitation and a concomitant decrease in Hawaiian precipitation.

Inspection of Fig. 5b reveals that both the western tropical Pacific-Indian Ocean SSTs and Mt. Hunter wintertime accumulation have continued to progressively increase since the 1976/77 PDO transition. This post-1977 increase contrasts with steady wintertime precipitation at south coastal Alaska weather stations following the precipitation jump in 1976/77 (Fig. 4E). This may indicate a heightened sensitivity to tropical SST teleconnections at higher elevation in Alaska. Other studies from the nearby St. Elias range have inferred a similar sensitivity of high elevation hydroclimate to conditions in the tropical Pacific Ocean^35,37^. The Rossby waves responsible for these tropical teleconnections are best defined in the free troposphere^43^, which may explain the preferential expression of tropical connections at high altitudes in Alaska. Additionally, mountain summit areas are more representative of free-air conditions compared to stations located in valleys or along the coast where local effects may mask the tropical climate signal^47^.

We examined output from seven CMIP5 GCM simulations (Table S1) to assess whether Last Millennium Experiment reconstructions capture the Aleutian Low intensification we infer from the Mt. Hunter record. All of the GCM simulations show warming in the western Pacific (15°S–15°N, 110°–160°E) with an ensemble average warming of 0.57 °C during the last two centuries comparing well with the 0.5° warming estimate from paleoclimate records^45^. In contrast, only 3 of the 7 models show significant (p < 0.05) deepening of the Aleutian Low (defined by the NPI index) (Fig. 6). None of the models match the observed increase in precipitation over the central Alaska Range or southern Alaska (58°–62°N, 135°–155°W), nor does the ensemble show a consistent precipitation decrease in Hawaii (Fig. S7). Those models that do suggest a precipitation increase in southern Alaska (MPI-ESM^48^ and IPSL-CM5A-LR^49^) produce a 5–10% rise in precipitation during the last two centuries, considerably less than shown in the Denali accumulation record. Furthermore, these two models are both among the simulations that generate an Aleutian Low deepening since 1840, and have the greatest increase in Western Pacific SSTs, although the SST increase in both models is significantly larger than in proxy reconstructions^45^. We suggest that current GCMs underestimate the sensitivity of North Pacific atmospheric teleconnections to tropical SSTs, and thus further model development focused on improving the extra-tropical responses to tropical SST warming may improve North Pacific hydroclimate projections in a warming world.

Although the future response of central-eastern tropical Pacific SSTs to increased radiative forcing remains unclear^50^, GCM simulations show enhanced ocean warming in the western tropical Pacific and Indian Ocean through the 21^st^ century in response to higher GHG concentrations^51,52^. The Mt. Hunter accumulation record corroborates and significantly extends instrumental (Fig. 4) and paleoclimate^15,16^ records showing precipitation increases in southern Alaska during the 20^th^ century. While hemispheric-scale reconstructions have yet to reveal coeval intensification of 20^th^ century hydroclimate anomalies outside of the North Pacific^41^, our data imply that regions such as Mt. Hunter, which are sensitive to tropical teleconnections, may continue to experience hydroclimate variability well outside the natural range of the past millennium.

## Methods

### Ice Core Collection and Processing

The Mt. Hunter ice cores were drilled in 2013 at 3,900 meters elevation from the saddle between the north and middle peaks of Mt. Hunter. Two 8 cm diameter cores were drilled to bedrock (208 m deep) using a Badger-Eclipse ice drill. After drilling, ice cores were shipped to the National Ice Core Laboratory in Denver, Colorado where the cores were weighed and measured for density calculations at ~1 m resolution, and cut into 3 × 3 cm sticks for melting at Dartmouth.

### Laboratory Procedures

The ice cores were continuously sampled on the Dartmouth Ice Core Melter system, which features an ultra-clean silicon carbide melt head that separates potentially contaminated meltwater from the outside of the core and pristine meltwater from the core interior^53^. The water from the interior fraction was pumped through an Abakus (Klotz) laser particle counter and particle size analyzer, a liquid conductivity meter (LCM) and finally into pre-cleaned vials for chemical analyses^54^. Denali core 2 was sampled and analyzed back to 190 m depth (800 CE), while only the top 133 m (1870 CE) were sampled and analyzed from core 1 to duplicate the critical instrumental period. Of the 190 meters of core 2 analyzed for this study, only 2.23 meters of ice had low core quality and excessive fracturing (170.58–171.68 m, 20 years; 179.70–180.11 m, 10 years; 181.56–181.72 m, 5 years; 185.02–185.44 m, 24 years; 187.76–187.88 m, 7 years; 189.50–189.59 m, 5 years) and could not be sampled using the continuous melter system. These sections were instead sampled discretely for stable water isotopes only.

Ice core melter samples were collected at a resolution averaging 5 cm, depending on melt rate, except that isotope samples were collected at ~1 cm resolution from 1750 to 1250 CE. LCM and particle concentration and size distribution were measured at an effective resolution of 1 millimeter. Major ion concentrations were measured on a Dionex 5000 capillary ion chromatograph (IC). Each IC analysis run of 50 samples included two sets of anion and cation standards for each analyte as well as four clean water blanks directly from the MilliQ filtration system, two blanks of MilliQ water from the bottle used to flush the lines in the melter system, and three blanks of MilliQ water that has been run through the melter head and the entire system. Water isotope ratios were measured by a Picarro L2130i Cavity Ring Down Spectrometer linked to a high throughput vaporizer at the University of Maine. Results are reported relative to SMOW (Standard Mean Ocean Water). Long-term precision is 0.1‰ (1σ) for δ^18^O and 0.4‰ (1σ) for δD, based on multiple analyses of internal and international standards.

### Ice Core Dating

To develop the Denali ice core timescale, three experienced researchers (D.W., E.O., D.F.) independently counted annual layers in the Mt. Hunter ice core chemical data. The timescale to 1777 CE was determined by counting annual oscillations in δ^18^O (summer peak), melt layers (summer peak), magnesium (spring peak), dust (spring peak), liquid conductivity (summer peak), ammonium (summer peak) and methanesulfonic acid (MSA; late summer-fall peak), consistent with previous North Pacific ice cores^16,21,35,55–57^. Using the relative peak positions of MSA in the late summer-fall and magnesium in the spring we distinguish cold-season (September-April) and warm-season (May-August) snow accumulation on Mt. Hunter back to 1867 CE. The timescale from 1777 to 1500 CE is based on annual oscillations of δ^18^O, δD, deuterium excess, dust and liquid conductivity measurements that were made at higher resolution than the other analytes, while conductivity and dust concentration measurements were exclusively used to date the ice core from 1500–800 CE.

The Mt. Hunter chronology is validated from 1750–2013 CE by comparing the timing of peaks in sulfate, chloride and conductivity to the known dates of explosive volcanic eruptions^53,58,59^ (Fig. S2). The volcanic events are used only as validation of our layer counting efforts and the timescale was not forced to match any particular event. There is no offset between our timescale and known volcanic eruptions as indicated by peaks in sulfate, chloride and conductivity during the 19^th^ and 20^th^ centuries, indicating a precision within +/−1 year throughout the last 200 years. The volcanoes in the 18^th^ century used for chronology validation (Laki, 1784 and Pavlof, 1763) were offset by one year from our chronology. Additionally, Cs-137 concentrations in the Mt. Hunter core strongly peak in 1963 during the largest atmospheric nuclear weapons testing campaign, which closely matches other published data^16,56^.

### Accumulation Uncertainty

Errors in the reported accumulation values (Y direction in Fig. 2) are due to three factors. First, there is uncertainty due to spatial and stochastic processes including wind redistribution, which we quantify using the difference in layer thicknesses between the primary and secondary ice cores. The layer thickness records from the two cores have a correlation coefficient of 0.92 and a slope of 0.96. During the last 100 years, the average percent difference in annual accumulation between the two cores is 10.2%, which we assume is consistent throughout the core and which we refer to as “stochastic error”.

There is a range of depths for each January 1 position because the three layer counters picked slightly different spots due to abnormal peak shapes, ambiguity, dispersion and human error. For each annual layer, the so-called “Peak Position Error” is quantified by dividing the range in January 1 position by the annual layer thickness. In cases where only two of the three counters picked a year, we assign an uncertainty of +/−50% of the annual layer thickness to that year. In cases where the core was damaged and equally spaced years were assigned, the uncertainty range is defined as the difference between the thinnest and thickest layers within 10 years of the gap. Peak position error is generally equal to about 10 cm (w. eq.) in the final thinning-corrected accumulation record, although it is occasionally larger in sections of low core quality when uncertainty assignments of +/−50% are more common.

As described in detail in the Supporting Information, we use four ice flow models to quantify the level of uncertainty in accumulation rate arising from the choice of flow model for layer thinning correction. For each year we define the “modeling error” as the entire range between the minimum and maximum model runs.

Stochastic error and peak position error are assumed to be symmetrically distributed around a single mean accumulation value and are aggregated using Eq. 1,

1 $${U}_{t}=\sqrt{\frac{{S}_{t}^{2}+{P}_{t}^{2}}{2}}$$

where U_t_ is the total uncertainty in year t, S_t_ is stochastic uncertainty and P_t_ is peak position uncertainty. We then define our upper and lower uncertainty bounds by adding U_t_ to the maximum uncertainty bound from our flow modeling simulations, and subtracting U_t_ from our minimum flow modeling uncertainty bound. This analysis shows that it is virtually certain that accumu lation on Mt. Hunter has never approached its present day values at any time in the last millennium.

### Analysis of Instrumental and Reanalysis Data

Alaska weather station data were obtained from the Global Historical Climatology Network^6^ and accessed through the Climate Change Institute Climate Reanalyzer. Months with missing precipitation data were filled with the average value for that month over the period of record. Reanalysis datasets were accessed through the Climate Change Institute Climate Reanalyzer website as well as the NCEP/NCAR monthly composites website. We use NCEP/NCAR 20^th^ century reanalysis data^27^ because of the long dataset duration. We restrict our analysis to the last 100 years (1910–2010) since both the resolution of our seasonal accumulation record and the reliability of the reanalysis data are higher after 1910.

### Change Point Detection

Following Abram *et al*.^44^, we define the point of sustained and significant increase in our time series as the point at which trends significant at p < 0.1 persist through present day. We used SiZer analysis with code from Abram *et al*.^44^ that was developed from Hannig and Marron^60^ and Chaudhury and Marron^61^. Using this technique, the average rate of accumulation change is calculated across moving windows ranging from 15 to 50 years, effectively removing high frequency variations from the timeseries to varying degrees. For Mt. Hunter, 1840 CE is the median date (among the different degrees of smoothing) at which all subsequent accumulation rates trends remain positive. Input data were annually sampled except for reconstructed Hawaii precipitation^46^, which is only available after 21-year smoothing. For consistency, analysis was conducted between 1625 CE and 2000 CE for each dataset.

### Global Climate Model Output

To compare our results with CMIP5 GCM simulations^48,49,62–66^, we obtained GCM output of sea level pressure and precipitation rate over the Alaska region over the duration of our study. GCM data were accessed through the Earth System Grid Federation data node https://pcmdi.llnl.gov/esgf-idp. There were seven GCMs available with data spanning the period from 1000–2005 CE and each of these is described in Table S1.

We calculated the average monthly sea level pressure values over the area from 160°E to 140°W and 30°N to 65°N in order to reconstruct the North Pacific Index (NPI) as defined in Trenberth and Hurrell^28^. We used GCM output from September-April to match our winter accumulation record. Sea surface temperature is not available across all seven GCM ensembles so we use surface air temperature from 15°S–15°N, 110°–160°E as an indicator of surface conditions over the Western Pacific warm pool. Results are standardized relative to the 1980–2000 mean. We extracted monthly precipitation rate output from the GCMs over the Western Pacific (15°S–15°N, 110°–160°E), Hawaii (15°–25°N, 155°–170°W), Southern Alaska (58°–62°N, 135°–155°W) and Mt. Hunter (63°N, 151°W), and combined the data into annual and seasonal averages (Fig. S7).

## Electronic supplementary material

Supplementary Information
