# Supplementary material for: Industrial-age doubling of snow accumulation in the Alaska Range linked to tropical ocean warming
Source: Sci Rep. 2017 Dec 19;7:17869. doi: 10.1038/s41598-017-18022-5 (PMC5736703; doi:10.1038/s41598-017-18022-5)
Supplement: Supplementary file 1 — Supplementary Information [file 41598_2017_18022_MOESM1_ESM.pdf]

Supplementary Information

Industrial-age doubling of snow accumulation in the Alaska Range linked to tropical ocean warming

Dominic Winski[1*], Erich Osterberg[1], David Ferris[1], Karl Kreutz[2], Cameron Wake[3], Seth Campbell[2], Robert Hawley[1], Samuel Roy[2], Sean Birkel[2], Douglas Introne[2], Mike Handley[2]

[1]Department of Earth Sciences, Dartmouth College, Hanover, NH 03755

[2]Climate Change Institute and School of Earth and Climate Sciences, University of Maine, Orono, Maine 04469

[3]Institute for the Study of Earth, Oceans, and Space, University of New Hampshire, Durham, NH 03824

*Correspondence to Dominic Winski. Email: dominic.a.winski.gr@dartmouth.edu

Timescale Uncertainty

We estimate the uncertainty in the timescale based on the level of agreement by three experienced layer counters (D.W., E.O., D.F.) tasked with picking the position of January 1 for every year. When reconciling the picks among the three layer counters, we identified clusters of picks while preserving the number of years picked by researchers within a given depth range (Fig. S1). Picks are considered to represent the same year if there are non-repeating picks clustering within +/- 0.5 years of each other (Fig. S1). If more than one pick for a given layer counter is present within this span, the pick that most closely agrees with the others is chosen.

For every depth in the core, there are four possibilities for the assignment of a January 1 "pick": 1) No one picks this depth; 2) one counter picks this depth while the other two do not; 3) two counters pick this depth while the other counter does not; and 4) all three counters pick this depth. For scenario #1, there is no pick at this depth in the final timescale. For scenario #2, the

timescale also does not include a pick at this depth. However, for uncertainty calculations, we allow a 50% chance that a January 1 could have been present there. For scenario #3, we assign a pick to this depth in the final depth-age scale. However, because there was disagreement, in uncertainty calculations we allow a 50% chance that this pick is erroneous and does not represent a January 1 position. For scenario #4, there is 100% chance of a January 1 pick there and the middle pick is chosen as the position of January 1. For each year, the spread in the position of January 1 is retained such that each year has an associated peak position error ranging from < +/- 1% up to +/- 50% of that year's accumulation. Within sections of the core that were damaged (170.58-171.68 m, 179.70-180.11 m, 181.56-181.72 m, 185.02-185.44 m, 187.76-187.88 m, 189.50-189.59 m), year spacing is interpolated at a spacing equal to the average of the ten years above and below the gap.

By 190 m depth, we find 184 instances where there may be an extra erroneous pick (scenario #3) and 191 instances where a pick may be missing (scenario #2), resulting in an age range of 619 CE to 994 CE at 190 m, with the most likely age of 810 CE. However, to reach either of these end members, all of the scenario #2 picks would have to be true and all of the scenario #3 picks would have to be false, or vice versa. Assuming the probabilities of missing and extra layers in scenarios #2 and #3, respectively are 50%, we take all possible combinations of each scenario quantified by a binomial distribution. Given no systematic bias toward under- or overcounting, the 99% confidence interval of these age distributions at the bottom of the dated section of the core (190 m) ranges from the year 780-830 CE. This error analysis neglects the possibility that all three layer pickers were systematically wrong in their layer choices in the same direction.

Prior to 1777 CE, we used the automated layer counting program, Straticounter[1], as a means to corroborate our manual layer counts. Straticounter closely matches our timescale to +/- 5 years at 1500 CE. Below this point, Straticounter consistently produces older ages (thinner layers) than the manual layer counts (Fig. S2), which would lead to a larger accumulation increase early in the record. We report the more conservative manual layer-counted accumulation data set, although the Straticounter timescale lies within the total range of uncertainty.

Flow modeling

From annual layer counting, we know the thickness of each year's accumulated snow extending back as far as annual layer counting has been performed (810 CE). However, there are a series of corrections that must be made in order to convert annual layer thickness to annual accumulation.

Various ice flow models have been developed to calculate the original thickness of layers before strain thinning due to compression and glacier flow. This is particularly important at Mt. Hunter because the record used for this work extends nearly the entire length of the core to bedrock. We use four independent modeling approaches to correct our annual layer thicknesses for thinning to assess the sensitivity of the final accumulation record to various modeling approaches: 1) We use a 3-dimensional finite element model developed by Campbell et al.[2] using field measurements of velocity, as well as surface and basal geometry; 2) we calculate accumulation rates by statistically optimizing three independent one-dimensional flow models (the Nye model[3], the Hooke model[4] and the Dansgaard-Johnsen (DJ) model[5]); 3) we build an additional, independent flow model based on field velocities and Glen's Flow Law (hereafter the

Hawley Model); and 4) we apply the suite of conditions tested by Thompson et al.[6] on Quelccaya for both Class I and Class II flow models. Each is described in more detail below.

The Campbell Model

The Campbell Model: The Campbell model is a 3-dimensional finite element glacier model developed using the COMSOL Multiphysics software package by Campbell et al[2]. The glacier geometry at the drill site is a saddle-shaped plateau measuring 1,000 meters north to south, 1,200 meters east to west and 200-250 meters in thickness, with very low slopes near the center of the divide. The detailed surface geometry and topography was measured using a high-precision Global Positioning System (GPS) field survey, while bed topography is based on gridded ground penetrating radar (GPR) surveys[2]. Both GPS and GPR surveys covered the plateau with a grid spacing of 150 meters, making this one of the most thoroughly characterized alpine ice core drill sites to date. Surface accumulation and velocity vary throughout the divide region but are also accounted for in the flow model based on GPR profiles and GPS velocity measurements, respectively. Although velocities across the plateau vary from near 0 meters/year near the divide to as much as 15 meters/year near the edge of the plateau, the drill site itself is located within 50 meters of the divide and has a measured flow velocity of 2 meters/year.

The model boundary conditions are constrained by the field measurements described above. All flow in the model is gravitationally driven and exits the glacier via icefalls to the east and west (mountains constrain the flow to the north and south). No-slip conditions are imposed on the glacier bed resulting from field measurements of a -17° C basal temperature. Within the model, Glen's Flow Law[7] is applied to simulate flow and ice rheology at the site. Ice viscocity values were determined using the power law method[2,8],

(S1) $$\mu = m\left(\frac{\partial \gamma}{\partial t}\right)^{n-1}$$

where μ is dynamic viscocity, γ is shear strain and n and m are constants optimized iteratively in the model to values of 0.333 and 6.5 **x** 10^{8} kg $s^{-\mathbf{n}}m^{-1}$, respectively, based on the dynamic viscosity range near the borehole approaching 1.0 x 10^{14} $Pa^{1}s^{1}$. Although these numbers correspond to the recommended A value of 1.2 **x** 10^{-25} $s^{-1}Pa^{-3}$ in Glen's flow law at –20°C and n = 3.0, which is widely used in glacial modeling studies[8], we ran the model multiple times using dynamic viscosities ranging from 8 x 10^{13} $Pa^{1}s^{1}$ to 1.5 x 10^{14} $Pa^{1}s^{1}$ in the vicinity of the borehole, corresponding to temperatures of -15° to -25° C. Although these conditions vary considerably more widely than would be expected given the geophysical data collected at the site, we use this range of model runs to conservatively assess our uncertainty in the vertical profile of glacier flow at the borehole.

For each model simulation, we extracted a vertical profile of strain rates at sub-meter resolution from the surface to bedrock at the specific borehole site. This strain rate profile was interpolated along the depth-age scale such that each year has an associated strain rate, ε_{zz}, in a^{-1}. We prescribe a strain rate equal to 0 at the top of the core where layers have a density less than 0.83 g/cm^3 with the assumption that compressive strain above this level leads to densification. Between densities of 0.83 and 0.9, we calculate the strain needed to produce the observed densification and subtract that strain from the vertical output profile. At layers greater than a density of 0.9 (depth = 61m, year = 1965), we apply all of the modeled strain to layer thinning. We integrate the resultant strain rate profile with respect to time (t) and calculate annual accumulation rates, b_t, by multiplying the cumulative strain at each annual layer (one minus the strain rate integral to be in the same coordinate system) by the observed thickness of the layer, λ_t:

(S2) $$b_t = \left(1 - \int_0^t (\varepsilon_{zz}) dt\right)\lambda_t$$

The result is the initial water-equivalent layer thickness for each annual layer at the surface, or annual accumulation (shown in Figs. 2-3). The annual accumulation time series is based on physical laws of glacier flow and does not rely on any assumptions of steady state accumulation.

The Hooke Model: Of the three 1-D depth-age models considered (the Nye model[3], the Hooke model[4] and the Dansgaard-Johnsen (DJ) model[5]), the Hooke model most closely reproduced the Mt. Hunter timescale, which is expected given that the Hooke model was developed for use in high relief areas[4]. In the Hooke model (equation S3),

(S3) Hooke $$a = \frac{H^m}{(m-1)b}\left[\frac{1}{(H-z)^{m-1}} - \frac{1}{H^{m-1}}\right]$$

a=age, H=total water equivalent thickness, z=water equivalent depth. The model is statistically optimized over its parameter space allowing for values of accumulation rate (b) and thinning parameter (m) to be selected that minimize the sum of the root mean square difference in depth between the modeled and counted depth for each year, by:

(S4) $$Error = \sum_0^a \sqrt{(z_m(a))^2 - (z_l(a))^2}$$

where z_m is the model depth and z_l is the layer count depth, both of which are functions of age (a). The

thinning parameter, *m,* is held constant over the whole core (at an optimized value m=1.92) but accumulation, *b,* is allowed to vary every 30 years given that a fixed accumulation rate is improbable at this site. The model results are insensitive to changes in the increment over which *b* changes. We tested increments of 10 to 100 years with no discernable change in the shape of the accumulation output. The resultant accumulation time series from the Hooke model is displayed in Fig. 3. Because Hooke's model is dependent on both the input timescale as well as our choice of m, we calculated accumulation values using a range of thinning parameters (*m* = 1.8-2.0) as well as the 99% confidence interval of our timescale (both old and young end members).

The Hawley Model: As an additional independent modeling approach, we use the Hawley Model to calculate a thinning function for accumulation layers due to ice flow. We first use the surface velocity field measured by GPS at the ice core site[14] and then scale the velocities with depth according to Glen's Flow Law[7]. We then use conservation of mass (equation S5), together with the divergence in the horizontal plane (equation S6), to determine the thinning as a function of depth:

(S5) $du/dx + dv/dy + dw/dz = 0$

(S6) $dw/dz = -(du/dx + dv/dy)$

This provides us with an instantaneous thinning function at any depth z. To find the total thinning for any given depth, we integrate *dw/dz* with respect to time, similar to the procedure in the finite element model. Since this modeling approach is reliant upon the accuracy of the velocity data, we run a Monte-Carlo simulation whereby model strain rates are derived using velocity data with a 2 σ uncertainty in

velocity (+/- 0.944 m/yr based on calculations in Campbell et al.[2]) randomly added or subtracted from each of the 38 points with measured velocities. We use the maximum and minimum strain rate profile among 1000 simulations to calculate the high and low bounds of thinning (Fig. S3) and accumulation using equation S2 (Fig. 3).

The Thompson Model: We use the Class I and Class II flow models described in Thompson et al. [6] for the characterization of Quelccaya Ice cap.

(S7) $$\varepsilon_{zz} = \varepsilon_0 \left(1 - \frac{z}{H}\right)^n$$

(S8) $$\varepsilon_{zz} = \varepsilon_0 \left(1 - \left[\frac{z}{H}\right]^n\right)$$

where ε_{zz} is the strain rate at depth z, ε_0 is the near surface vertical strain rate, z is depth (m w. eq.), H is the bed depth and n is a unitless exponent.

To apply these models to Mt. Hunter we calculate the strain profiles using a range of ε_0 from 0.0145 to 0.0275 a^{-1} corresponding to the minimum and maximum strain rate at the top of the glacier as calculated by the Campbell model. We then generate strain profiles using both Class I and Class II models for values of n ranging from 1 to 4 (as described by Thompson et al. [6]). For both Class I and Class II models we run 1000 simulations which randomly select values of n and ε_0 within the allocated parameter space, resulting in 2000 total simulations. The very large range of n and the use of both Class I and II models yield a substantially larger variation in thinning rates than the other models described above. However, nearly all of these models underestimate thinning with respect to the Campbell, Hooke and Hawley models (Fig. S3),

yielding greater accumulation trends in recent centuries compared with those in the Campbell, Hooke and Hawley models.

Having developed four separate modeling approaches to quantifying layer thinning, each with its associated uncertainty, allows us to aggregate the total model uncertainty for each year. Modeling uncertainty is defined as the range between the highest and lowest accumulation values among the thinning models.

Supporting Figures and Tables

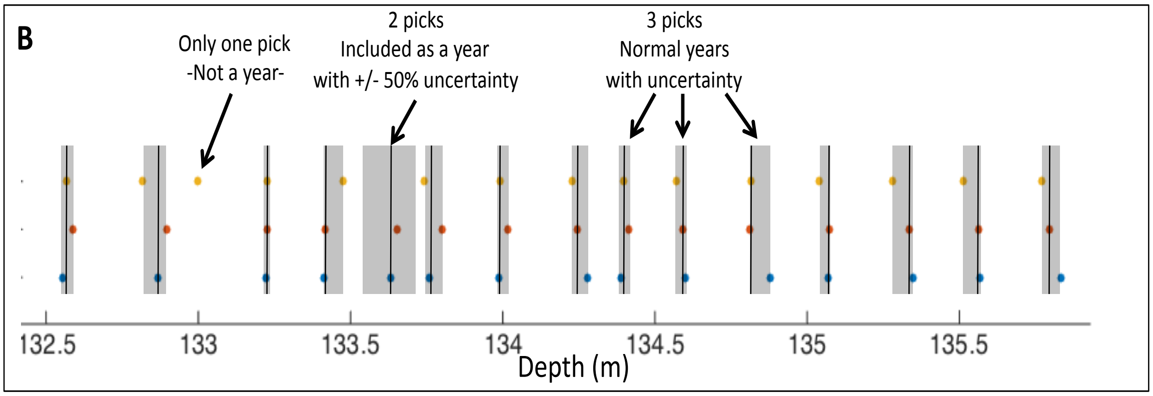

Fig. S1. B) Reconciling annual layer picks among counters. The yellow, blue and red dots indicate the Jan. 1 pick position of a particular researcher. The vertical black lines denote the position used in the depth-age scale and the gray shading indicates the peak position uncertainty.

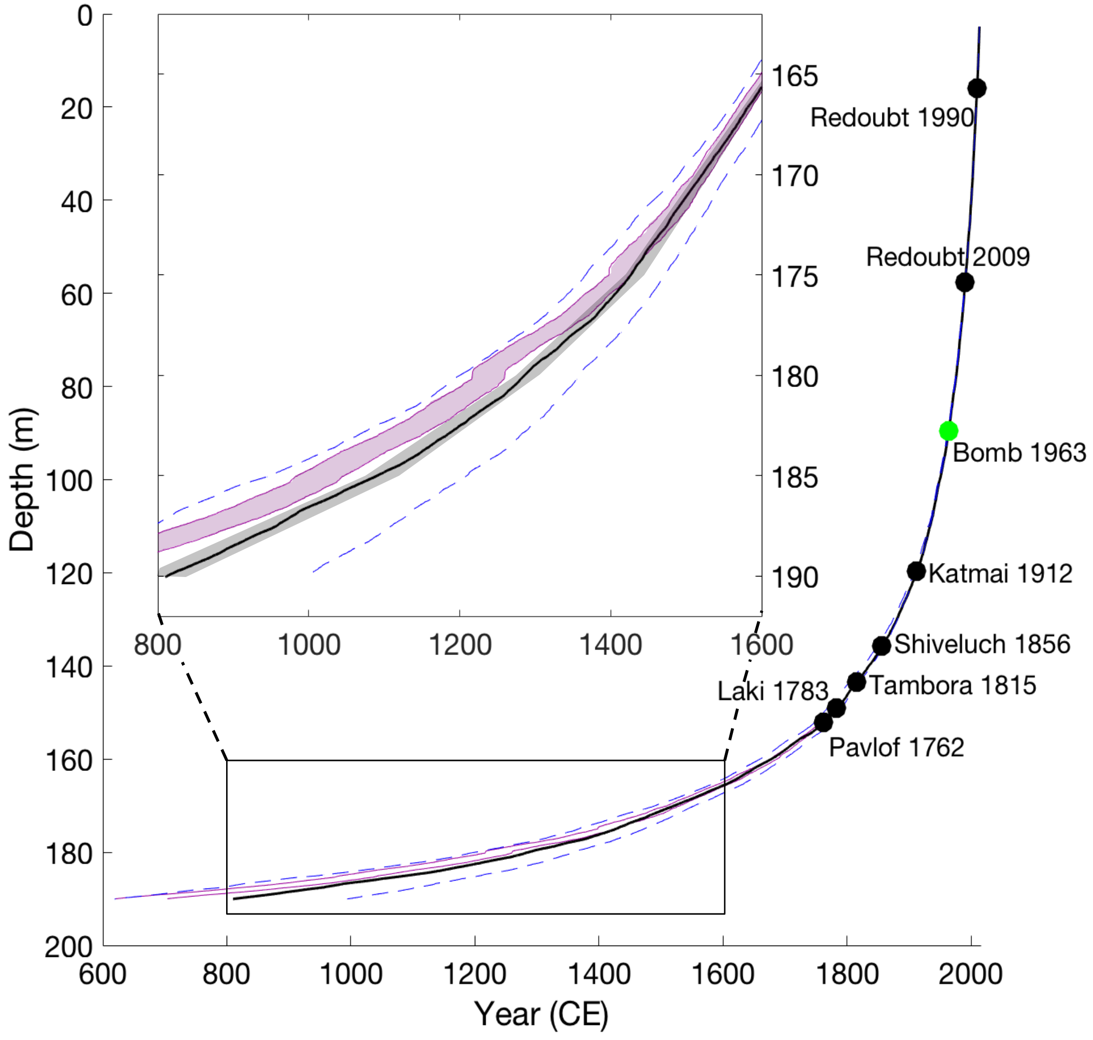

Fig. S2. The Mt. Hunter depth age scale. The black line in the center is the depth/age curve defined by consensus among layer counts between Ferris, Osterberg and Winski. The gray shading around the black line encapsulates the 99% confidence limits. The two blue lines represent the maximum layer counting error due to layer counting. The purple shading encompasses the upper and lower 95% confidence intervals of the Straticounter layer counting results. The Straticounter output agrees precisely (+/- 5 years) with the layer count depth/age scale until 1500. Below this point Straticounter produces older ages than layer counting efforts. Volcanic events identified in the core are marked with black circles. The Cs-137 peak at 1963 is marked by a green circle.

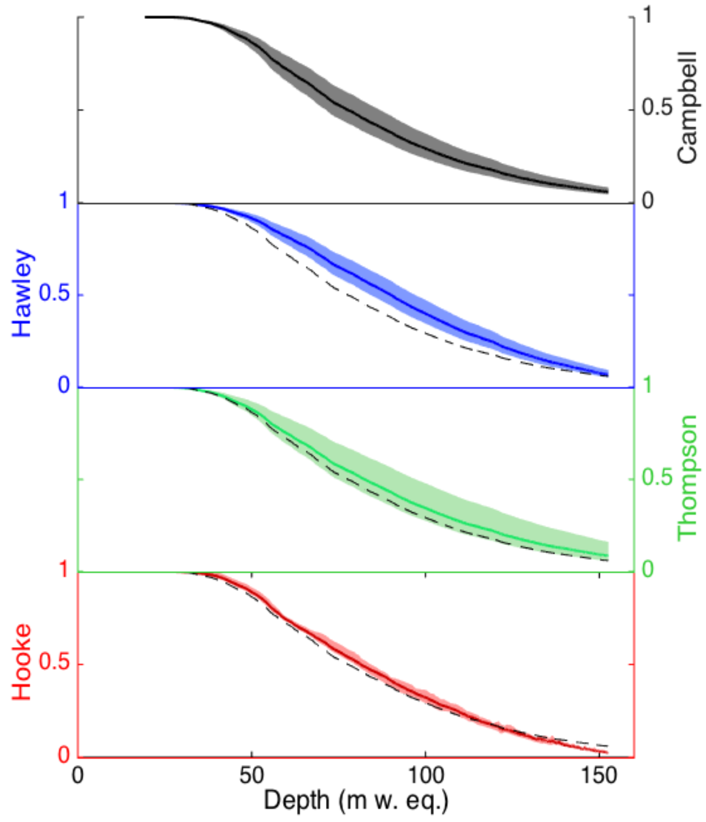

Fig. S3. Thinning functions from four flow models. Plotted above are ratios of observed layer thickness over original layer thickness from each of the four models with respect to depth. The mean estimate of each model [Campbell (black), Hawley (blue), Thompson (green) and Hooke (red)] is shown as a solid line with the entire range of thinning represented by shading. The thinning function from the Campbell model is shown on the bottom three plots as a dashed line for comparison. The Hawley and Thompson models estimate less thinning than the Campbell model throughout the profile, leading to greater rises in 19th-20th century accumulation in the Hawley and Thompson models (see Fig. 3). The Hooke model also estimates less thinning than the Campbell model above 120 meters, but estimates slightly more thinning than the Campbell model below this point. All four models are in agreement with the most rapid rates of thinning occurring at intermediate depths.

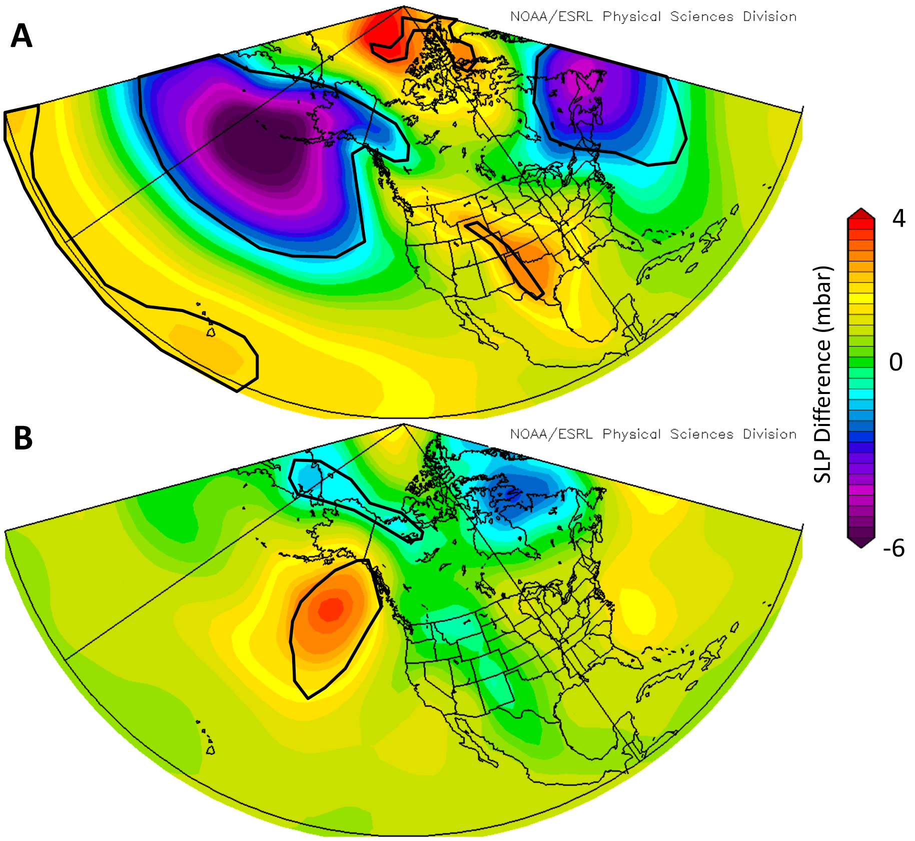

Fig. S4. Sea level pressure anomalies during extreme high and low accumulation winters. The maps above show sea level pressure differences (mbar) during the 10% highest and lowest accumulation winters (A) and highest and lowest accumulation summers (B) on Mt. Hunter during the NCEP/NCAR 20th century reanalysis[9] period (1871-2011). Areas significant to 95% are highlighted in bold. Results show that there is a deeper Aleutian Low during high accumulation winters (defined as September through April) on Mt. Hunter and a shallow Aleutian Low during low accumulation winters. Images obtained using the NCEP/NCAR monthly composites page (https://www.esrl.noaa.gov/psd/cgi-bin/data/composites).

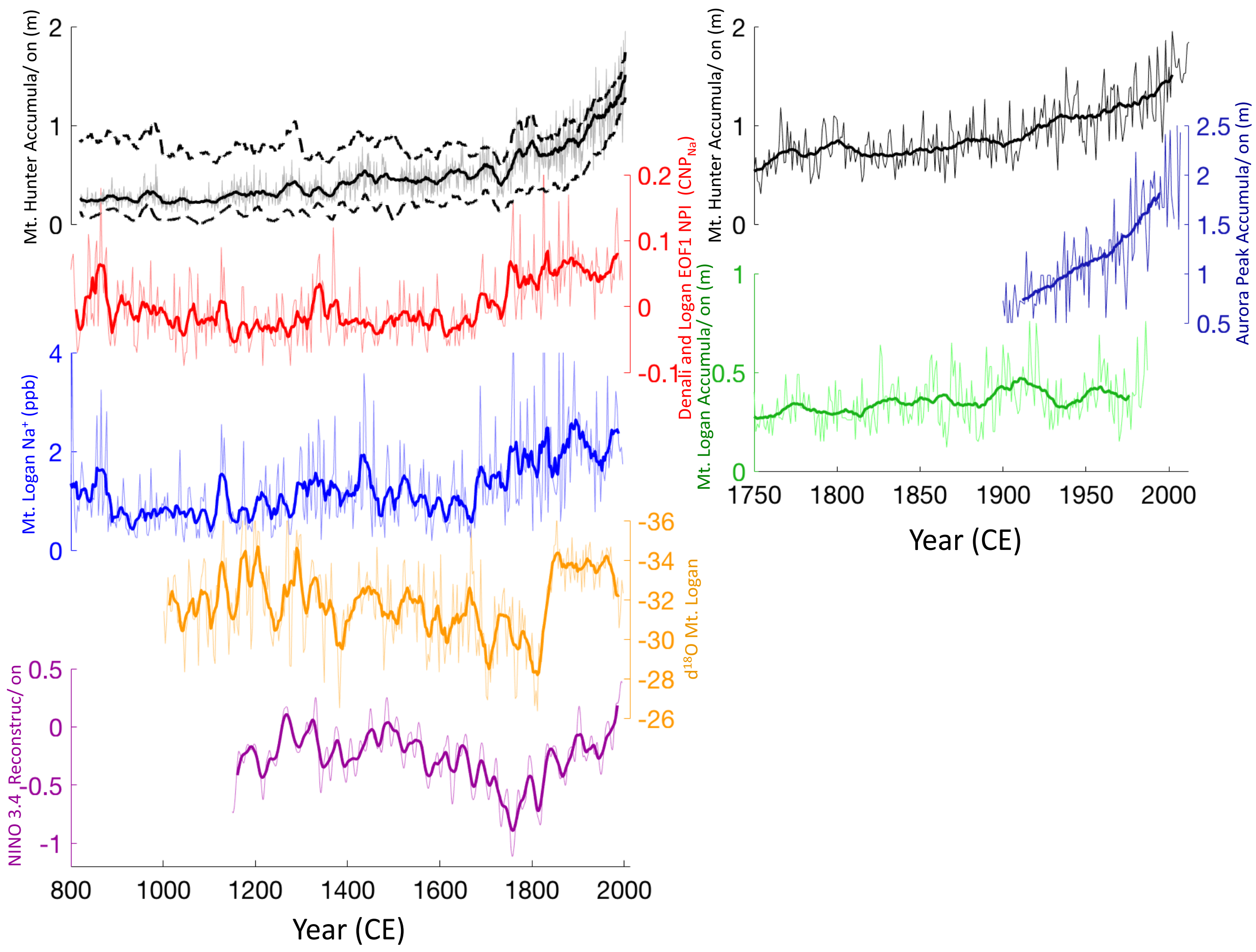

Fig. S5. Comparison with paleoclimate records. The Mt. Hunter annual accumulation time series (gray) with a 21-running mean (black - solid) and uncertainties (black – dashed) is compared with the Denali and Mt. Logan composite Na^+ record[10] (red), the Mt. Logan Na^+ record[11] (blue), and the Mt. Logan d^{18}O record[12] (orange), calibrated as a proxy for Aleutian Low strength. We include a reconstructed NINO3.4 index[13] (purple) for comparison. The inset at right shows the raw and 21-year smoothed accumulation time series of Mt. Hunter (black), Aurora Peak[14] (blue) and Mt. Logan accumulation[15] (green) since 1750.

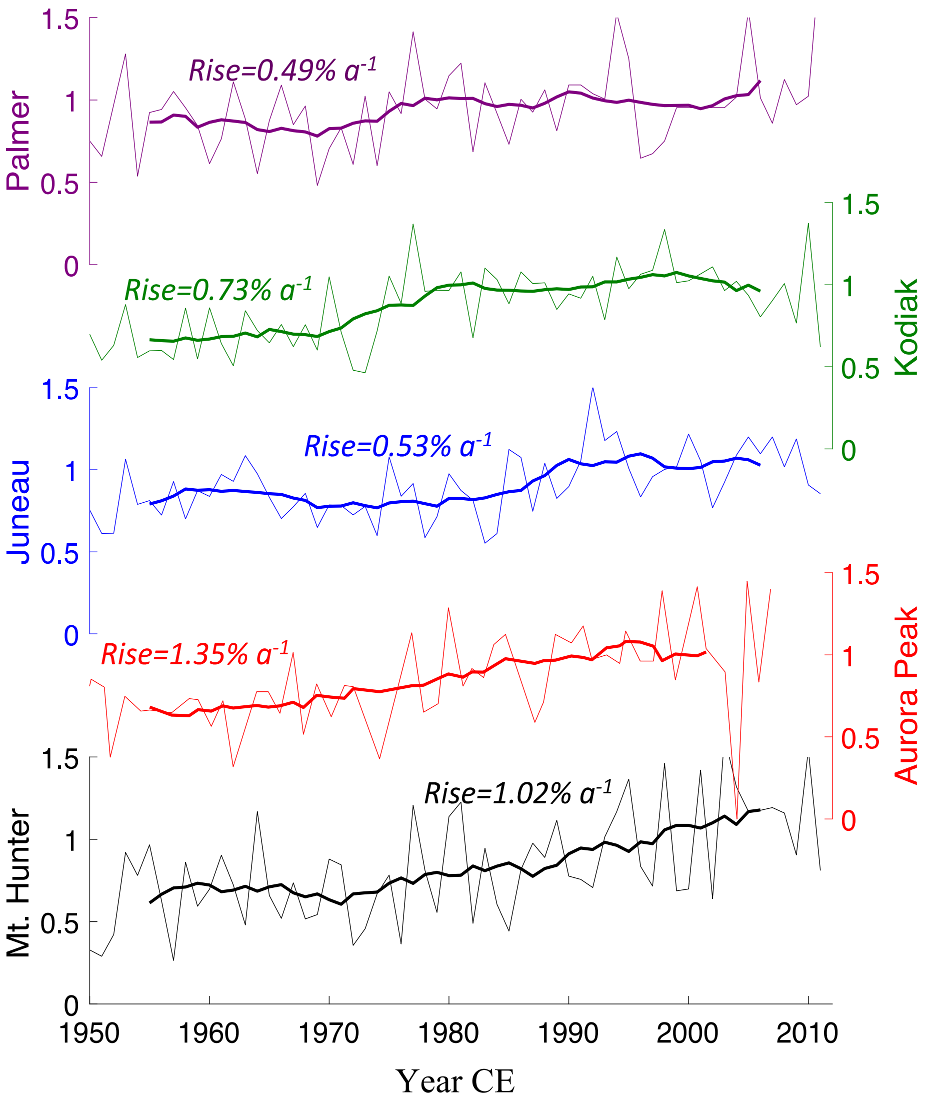

Fig S6: Comparison with instrumental period records. The Mt. Hunter accumulation time series since 1950 CE (black) is compared with accumulation at Aurora Peak (red) and winter (September-April) precipitation at Juneau (blue), Kodiak (green) and Palmer (purple). Bold lines indicate the 11-year running mean. Precipitation amounts are displayed relative to the mean values for 1980-2010. The mean rates of increase in precipitation since 1950 CE are labeled by each record.

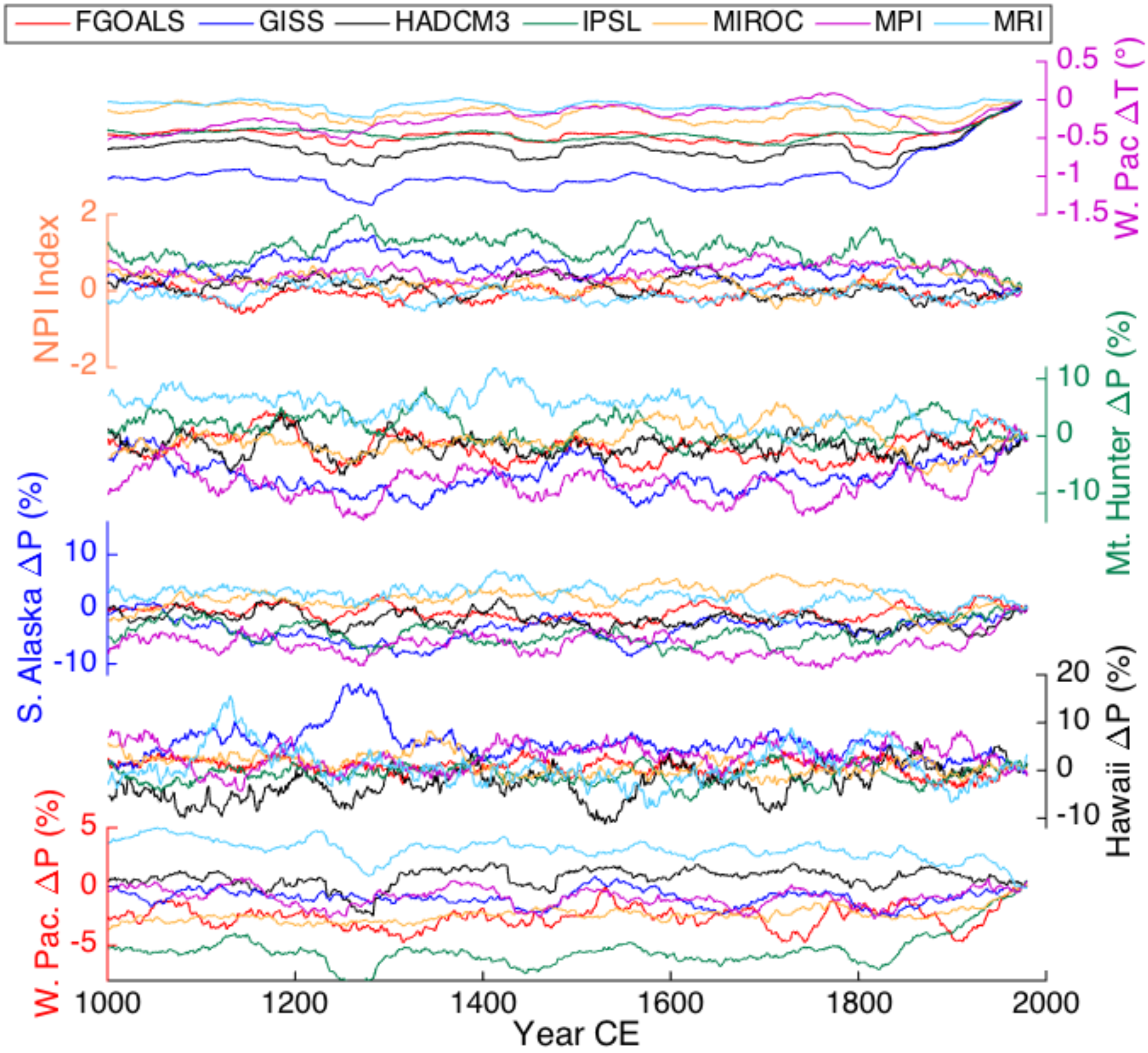

Fig. S7. GCM precipitation in the last millennium. Simulations of precipitation over the last millennium by seven global climate models in CMIP5 are shown as precipitation amount relative to the 1950-2000 mean. Results are smoothed with a 51-year running mean. For comparison, GCM derived western Pacific surface temperature and NPI index are shown at top. There is little consensus for directional changes in precipitation over the warm pool, Hawaii, coastal Alaska or over Mt. Hunter.

Model	Variable	Data Files		Ref.
		Past1000 (850-1850)	Historical (1850-2005)	
HadCM3	Precipitation	pr_Amon_HadCM3_past1000_r1i1p1_085001-185012.nc	pr_Amon_HadCM3_historical_r1i1p1_185912-200512.nc	(17)
	Sea Level Pressure	psl_Amon_HadCM3_past1000_r1i1p1_085001-185012.nc	psl_Amon_HadCM3_historical_r1i1p1_185912-200512.nc	
	Surface Air Temperature	tas_Amon_HadCM3_past1000_r1i1p1_085001-185012.nc	tas_Amon_HadCM3_historical_r1i1p1_185912-200511.nc	
MPI-ESM	Precipitation	pr_Amon_MPI-ESM-P_past1000_r1i1p1_085001-184912.nc	pr_Amon_MPI-ESM-P_historical_r1i1p1_185001-200512.nc	(20)
	Sea Level Pressure	psl_Amon_MPI-ESM-P_past1000_r1i1p1_085001-184912.nc	psl_Amon_MPI-ESM-P_historical_r1i1p1_185001-200512.nc	
	Surface Air Temperature	tas_Amon_MPI-ESM-P_past1000_r1i1p1_085001-184912.nc	tas_Amon_MPI-ESM-P_historical_r1i1p1_185001-200512.nc	
GISS-E2-R	Precipitation	pr_Amon_GISS-E2-R_past1000_r1i1p1221_085001-185012.nc	pr_Amon_GISS-E2-R_historicalMisc_r1i1p1221_185001-200512.nc	(19)
	Sea Level Pressure	psl_Amon_GISS-E2-R_past1000_r1i1p1221_085001-185012.nc	psl_Amon_GISS-E2-R_historical_r1i1p1_185001-200512.nc	
	Surface Air Temperature	tas_Amon_GISS-E2-R_past1000_r1i1p121_085001-185012.nc	tas_Amon_GISS-E2-R_historical_r1i1p121_185101-200512.nc	
FGOALS-gl	Precipitation	pr_Amon_FGOALS-gl_past1000_r1i1p1_100001-199912.nc	-	(18)
	Sea Level Pressure	psl_Amon_FGOALS-gl_past1000_r1i1p1_100001-199912.nc	-	
	Surface Air Temperature	tas_Amon_FGOALS-gl_past1000_r1i1p1_100001-199912.nc	-	
MRI-CGCM3	Precipitation	pr_Amon_MRI-CGCM3_past1000_r1i1p1_085001-184912.nc	pr_Amon_MRI-CGCM3_historical_r1i1p1_185001-200512.nc	(21)
	Sea Level Pressure	psl_Amon_MRI-CGCM3_past1000_r1i1p1_085001-184912.nc	psl_Amon_MRI-CGCM3_historical_r1i1p1_185001-200512.nc	
	Surface Air Temperature	tas_Amon_MRI-CGCM3_past1000_r1i1p1_085001-184912.nc	tas_Amon_MRI-CGCM3_historical_r1i1p1_185001-200512.nc	
IPSL-CM5A-LR	Precipitation	pr_Amon_IPSL-CM5A-LR_past1000_r1i1p1_085001-184912.nc	pr_Amon_IPSL-CM5A-LR_historical_r1i1p1_185001-200512.nc	(22)
	Sea Level Pressure	psl_Amon_IPSL-CM5A-LR_past1000_r1i1p1_085001-185012.nc	psl_Amon_IPSL-CM5A-LR_historical_r1i1p1_185001-200512.nc	
	Surface Air Temperature	tas_Amon_IPSL-CM5A-LR_past1000_r1i1p1_085001-184912.nc	tas_Amon_IPSL-CM5A-LR_historical_r1i1p1_185001-200512.nc	
MIROC-ESM	Precipitation	pr_Amon_MIROC-ESM_past1000_r1i1p1_085001-184912.nc	pr_Amon_MIROC-ESM_historical_r1i1p1_185001-200512.nc	(16)
	Sea Level Pressure	psl_Amon_MIROC-ESM_past1000_r1i1p1_085001-184912.nc	psl_Amon_MIROC-ESM_historical_r1i1p1_185001-200512.nc	
	Surface Air Temperature	tas_Amon_MIROC-ESM_past1000_r1i1p1_085001-184912.nc	tas_Amon_MIROC-ESM_historical_r1i1p1_185001-200512.nc	

Table S1. Table of the general circulation model simulations used in this paper[16-22].

1 Winstrup, M. *et al.* An automated approach for annual layer counting in ice cores. *Clim. Past* **8**, 1881-1895, doi:10.5194/cp-8-1881-2012 (2012).
2 Campbell, S. *et al.* Strain-rate estimates for crevasse formation at an alpine ice divide: Mount Hunter, Alaska. *Ann. Glaciol.* **54**, 200-208, doi:10.3189/2013AoG63A266 (2013).
3 Nye, J. F. Correction factor for accumulation measured by the thickness of the annual layers in an ice sheet. *j. Glaciol* **4**, 785-788 (1963).
4 Kaspari, S. *et al.* Snow accumulation rate on Qomolangma (Mount Everest), Himalaya: synchroneity with sites across the Tibetan Plateau on 50–100 year timescales. *J. Glaciol.* **54**, 343-352 (2008).
5 Dansgaard, W. & Johnsen, S. J. A flow model and a time scale for the ice core from Camp Century, Greenland. *J. Glaciol.* **8**, 215-223 (1969).
6 Thompson, L. G. *et al.* Geophysical investigations of the tropical Quelccaya ice cap, Peru. *J. Glaciol.* **28**, 57-69 (1982).
7 Glen, J. W. The flow law of ice: A discussion of the assumptions made in glacier theory, their experimental foundations and consequences. *IASH Publ* **47**, 171-183 (1958).
8 Cuffey, K. M. & Paterson, W. S. B. *The physics of glaciers*. (Academic Press, 2010).
9 Compo, G. P. *et al.* The twentieth century reanalysis project. *Q. J. R. Meteorol. Soc.* **137**, 1-28 (2011).
10 Osterberg, E. C. *et al.* 1200-Year Composite Ice Core Record of Aleutian Low Intensification. *Geophys. Res. Lett.* (2017).
11 Osterberg, E. C. *et al.* Mount Logan ice core record of tropical and solar influences on Aleutian Low variability: 500–1998 AD. *Journal of Geophysical Research: Atmospheres* **119**, 11,189-111,204 (2014).
12 Fisher, D. A. *et al.* The Mt Logan Holocene-late Wisconsinan isotope record: Tropical Pacific-Yukon connections. *Holocene* **18**, 667-677, doi:10.1177/0959683608092236 (2008).
13 Emile-Geay, J., Cobb, K. M., Mann, M. E. & Wittenberg, A. T. Estimating central equatorial Pacific SST variability over the past millennium. Part II: Reconstructions and implications. *Journal of Climate* **26**, 2329-2352 (2013).
14 Tsushima, A. *et al.* Reconstruction of recent climate change in Alaska from the Aurora Peak ice core, central Alaska. *Clim. Past* **11**, 217-226, doi:10.5194/cp-11-217-2015 (2015).
15 Moore, G. W. K., Holdsworth, G. & Alverson, K. Climate change in the North Pacific region over the past three centuries. *Nature* **420**, 401-403, doi:10.1038/nature01229 (2002).
16 Watanabe, S. *et al.* MIROC-ESM 2010: model description and basic results of CMIP5-20c3m experiments. *Geosci. Model Dev.* **4**, 845-872, doi:10.5194/gmd-4-845-2011 (2011).
17 Collins, M., Tett, S. F. B. & Cooper, C. The internal climate variability of HadCM3, a version of the Hadley Centre coupled model without flux adjustments. *Clim. Dyn.* **17**, 61-81, doi:10.1007/s003820000094 (2001).
18 Zhou, T. J., Li, B., Man, W. M., Zhang, L. X. & Zhang, J. A comparison of the Medieval Warm Period, Little Ice Age and 20th century warming simulated by the FGOALS

climate system model. *Chinese Science Bulletin* **56**, 3028-3041, doi:10.1007/s11434-011-4641-6 (2011).
19 Schmidt, G. A. *et al.* Present-day atmospheric simulations using GISS ModelE: Comparison to in situ, satellite, and reanalysis data. *Journal of Climate* **19**, 153-192 (2006).
20 Giorgetta, M. A. *et al.* The atmospheric general circulation model ECHAM6-model description. (2013).
21 Yukimoto, S. *et al.* A new global climate model of the Meteorological Research Institute: MRI-CGCM3—model description and basic performance—. *Journal of the Meteorological Society of Japan. Ser. II* **90**, 23-64 (2012).
22 Dufresne, J. L. *et al.* Climate change projections using the IPSL-CM5 Earth System Model: from CMIP3 to CMIP5. *Clim. Dyn.* **40**, 2123-2165, doi:10.1007/s00382-012-1636-1 (2013).
